# Changes in Dietary Total and Nonheme Iron Intake Is Associated With Incident Frailty in Older Men: The Concord Health and Aging in Men Project

**DOI:** 10.1093/gerona/glac077

**Published:** 2022-03-30

**Authors:** Rebecca Luong, Rosilene V Ribeiro, Anna Rangan, Vasi Naganathan, Fiona Blyth, Louise M Waite, David J Handelsman, Robert G Cumming, David G Le Couteur, Vasant Hirani

**Affiliations:** Nutrition and Dietetics Group, Sydney Nursing School, Faculty of Medicine and Health, The University of Sydney, NSW, Australia; ARC Centre of Excellence in Population Ageing Research (CEPAR), The University of Sydney, NSW, Australia; Charles Perkins Centre, The University of Sydney, NSW, Australia; School of Life and Environmental Sciences, Faculty of Science, The University of Sydney, NSW, Australia; Nutrition and Dietetics Group, Sydney Nursing School, Faculty of Medicine and Health, The University of Sydney, NSW, Australia; Charles Perkins Centre, The University of Sydney, NSW, Australia; Centre for Education and Research on Ageing, Concord Hospital, The University of Sydney, Concord, NSW, Australia; Concord Clinical School, Faculty of Medicine and Health, The University of Sydney, Concord, NSW, Australia; ARC Centre of Excellence in Population Ageing Research (CEPAR), The University of Sydney, NSW, Australia; School of Public Health, The University of Sydney, NSW, Australia; Centre for Education and Research on Ageing, Concord Hospital, The University of Sydney, Concord, NSW, Australia; Concord Clinical School, Faculty of Medicine and Health, The University of Sydney, Concord, NSW, Australia; ANZAC Research Institute, The University of Sydney and Concord Hospital, Concord, NSW, Australia; ARC Centre of Excellence in Population Ageing Research (CEPAR), The University of Sydney, NSW, Australia; School of Public Health, The University of Sydney, NSW, Australia; Charles Perkins Centre, The University of Sydney, NSW, Australia; ANZAC Research Institute, The University of Sydney and Concord Hospital, Concord, NSW, Australia; Nutrition and Dietetics Group, Sydney Nursing School, Faculty of Medicine and Health, The University of Sydney, NSW, Australia; ARC Centre of Excellence in Population Ageing Research (CEPAR), The University of Sydney, NSW, Australia

**Keywords:** Diet, Dietary iron, Food, Frailty syndrome, Old men

## Abstract

**Background:**

Nutritional intake could influence the development of frailty. The aim was to evaluate the associations between dietary iron intakes and changes in dietary iron intakes with frailty.

**Methods:**

Cross-sectional analyses involved 785 men with Fried frailty phenotype (FP) and 758 men with Rockwood frailty index (FI) data aged 75 years and older at nutrition assessment from the Concord Health and Ageing in Men Project prospective cohort study. Of these, 563 men who were FP robust or prefrail, and 432 men who were FI nonfrail were included in the longitudinal analyses for more than 3 years. Dietary intake was assessed at both timepoints using a validated diet history questionnaire. The dietary calculation was used to derive heme iron and nonheme iron intakes from total iron intakes. The associations were evaluated through binary logistic regression.

**Results:**

Incidence of FP frailty was 15.3% (*n* = 86). In longitudinal analyses, maintaining total iron intakes (medium tertile −2.61–0.81 mg/d), increases in total iron and nonheme iron intakes (high tertiles ≥0.82 mg/d and ≥0.80 mg/d), and changes in nonheme iron intake (1 mg increment) were associated with reduced risks of incident FP frailty (OR: 0.47 [95% confindence interval (CI): 0.24, 0.93, *p* = .031], OR 0.48 [95% CI: 0.23, 0.99, *p* = .048], OR 0.41 [95% CI: 0.20, 0.88, *p* = .022], and OR 0.89 [95% CI: 0.82, 0.98, *p* = .017]).

**Conclusion:**

Maintaining or increases in total dietary iron and increases or changes in dietary nonheme iron intakes more than 3 years were associated with reduced incidence of FP frailty in older men.

Frailty has a higher prevalence in old age and has been defined as a state of increased vulnerability to adverse health outcomes secondary to multiple deficits in physiological, physical, and mental function (1). Older adults are susceptible to poor nutritional intake which is considered a key contributor to the development of frailty (2,3). Dietary total iron, heme iron, and nonheme iron intakes have been shown to decrease with increasing age in older adults (4,5).

Healthy dietary patterns containing nonheme iron sources have been associated with reduced risks of frailty (6), whilst protein-rich dietary patterns containing heme iron sources and animal protein have shown conflicting associations with frailty (7,8). Hence, the different forms of dietary iron intake (heme iron and nonheme iron) could have different effects on frailty.

The Concord Health and Ageing in Men Project (CHAMP) is a prospective cohort study examining the causes and consequences of major geriatric syndromes. A total of 1 705 men aged 70 years and older were recruited in the first wave (between January 2005 and June 2007) (9). Dietary data collection were added at 5-year follow-up of the CHAMP study with 794 men aged 75 years and older (10). The association between anemia and frailty was previously investigated in the CHAMP cohort, which found that older men with anemia had increased risks of frailty in both cross-sectional and longitudinal analyses (11). This was similarly shown in other studies that demonstrated that hemoglobin levels have an inverse dose-response relationship with frailty (12–15). Iron deficiency is one of the most common causes of anemia (11). However, research has also shown that iron deficiency, irrespective of anemia, has detrimental effects on physical capacity (16).

The direct associations between dietary iron intakes and frailty have not been previously examined. The aim of the present study was to investigate the associations between dietary iron intakes (total iron, heme iron, nonheme iron, and heme to nonheme iron ratio) and changes in dietary iron intakes with frailty, involving both cross-sectional and longitudinal analyses in older men from the CHAMP cohort.

## Methods

### Study Participants

**Figure 1.**
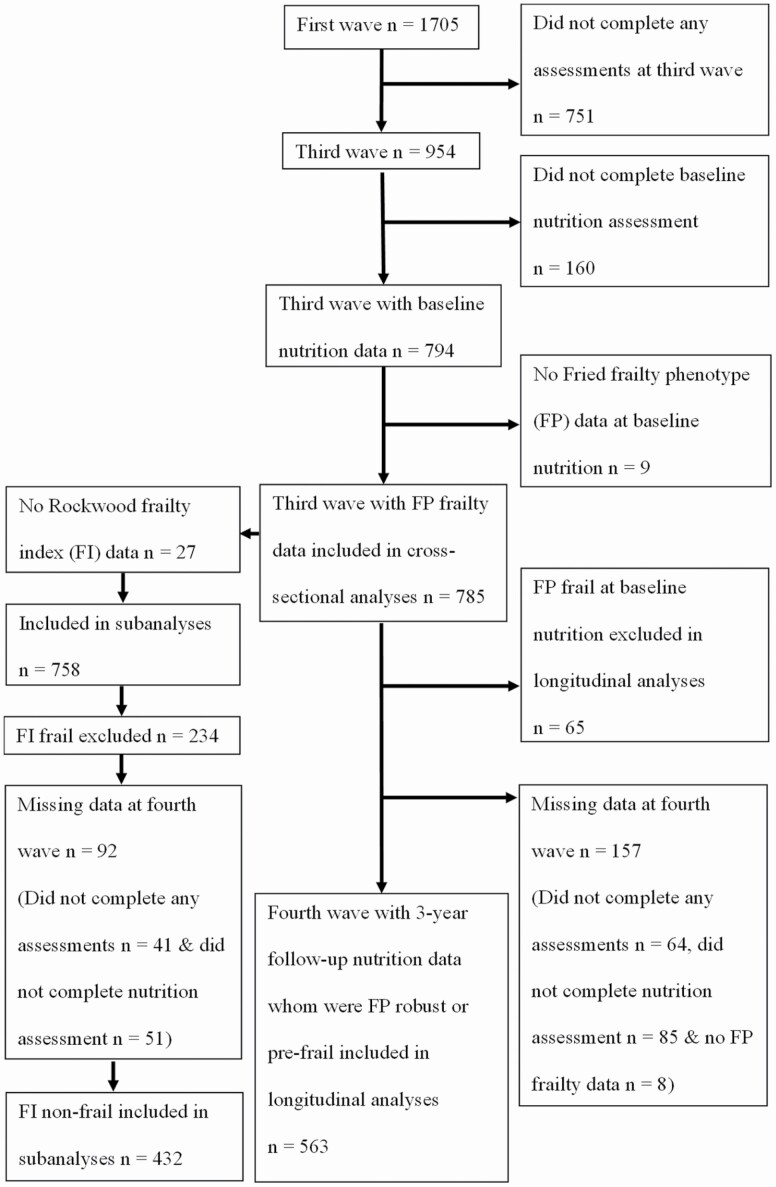
Flow diagram of participants included in cross-sectional and longitudinal analyses.

Dietary data were first collected in the third wave of CHAMP (between August 2010 and August 2013) involving 794 men aged 75 years and older (baseline nutrition). This was followed by nutrition data collection in the fourth wave of CHAMP (between August 2014 and June 2016) with 718 men aged 78 years and older (3-year follow-up in this study) (17). At baseline nutrition, 785 men (99%) also had Fried frailty phenotype (FP) data and were included in the cross-sectional analyses. Of these, 758 men (95%) had Rockwood frailty index (FI) data whom were included in subanalyses. Men who were FP frail and FI frail at baseline nutrition were excluded from longitudinal analyses. Of the 720 men who were FP robust or FP prefrail, 563 men (78%) had FP frailty and dietary data at 3-year follow-up and were included in the longitudinal analyses. Of the 524 men who were FI nonfrail, 432 men (82%) had FI frailty and dietary data at 3-year follow-up and were included in longitudinal analyses. Flowchart of participants’ inclusion in cross-sectional and longitudinal analyses is shown in Figure 1. The CHAMP study was approved by the Concord Hospital Human Research Ethics Committee (HREC/14/CRGH/17), and study participants provided written informed consent for all assessments.

### Dietary Intake

Dietary data were collected (at baseline nutrition and at 3-year follow-up) using a validated diet history questionnaire, involving questions about intake in the previous 3 months and used food models, photographs, and household measures to estimate amounts consumed (10,18). Entries were converted to foods, food groups, and total iron intakes using FoodWorks 7 Professional for Windows (Xyris Software (Brisbane, Australia) Pty Ltd) and The Australian Food, Supplement and Nutrient Database 2007 (AUSNUT 2007). Food groups included fruits, vegetables, grains, dairy/alternatives, and meat/alternatives.

Heme iron and nonheme iron intakes are not available in AUSNUT 2007. Thus, the dietary calculation was used to derive heme iron and nonheme iron intakes from total iron intakes (19). The average proportions of heme iron content in the Australian food supply published by Rangan et al. (19), for different food sources were used: pork (65%), poultry (62%), beef and lamb (61%), seafood (40%), and offal (33%). Heme iron intake from a single food source was calculated as the amount of the food source consumed multiplied by the proportion of heme iron content in the food source. Heme iron intake from a single food source in a recipe was calculated as the amount of the food source consumed multiplied by the proportion of the single food source contributing to the total iron of the recipe (excluding ingredient sources not containing any iron such as canola oil and white sugar) multiplied by the proportion of heme iron content in the food source. Heme iron intake from a recipe was calculated as the sum of the heme iron from all food sources containing heme iron in the recipe. Nonheme iron intake from a single food source or recipe was calculated as the total iron intake minus heme iron intake. Iron in all other food sources and recipes not containing heme iron were assumed to contain 100% of total iron from nonheme iron. The top sources of total iron, heme iron, and nonheme iron, as indicated by the sum of intakes of the top 5 individual food items from all participants in longitudinal analyses at baseline nutrition and at 3-year follow-up were identified. Changes in daily iron intakes were defined as mean intake at 3-year follow-up minus mean intake at baseline nutrition. Detailed data on dosage or levels of dietary supplements were not available, and thus iron intake from vitamin or mineral supplements were excluded in the analyses.

### Frailty Measurement

Frailty was defined using the FP criteria according to the Cardiovascular Health Study (CHS) for weakness and slowness (20), and adapted criteria for weight loss, exhaustion, and low activity due to unavailability of the exact measurement (11). Participants were classified as robust if they had none, prefrail if they had 1 or 2, and frail if they had 3 or more of the following FP components: weakness, slowness, weight loss, exhaustion, and low activity. Weakness was defined as being in the lowest sample quintile for grip strength adjusted for body mass index (BMI), measured by the mean value of 2 trials on each side using a Jamar dynamometer. Slowness was defined as being in the lowest sample quintile for walking speed adjusted for height, measured as the mean value of 2 trials on a 6-meter course at usual pace. Weight loss was indicated if a participant’s current weight (measured at the clinic visit) was lower by 15% or more than the self-reported heaviest (or lower than weight at 25 years old, if missing data on heaviest weight). Exhaustion was defined by participants reporting either “a little of the time” or “none of the time” on the Short-form Health Survey on “how much of the time during that past 4 weeks did you have a lot of energy?” (21). Low physical activity was indicated as being in the lowest sample quintile of activity as measured by the Physical Activity Scale for the Elderly (PASE; a score of 72 or lower among CHAMP participants) (22).

In subanalyses, frailty was determined by calculating the FI using 29 items as used in a previous analysis of CHAMP data (23), which was a modification of the FI (24). Deficits assessed included chronic medical conditions, disability, and symptoms. FI was calculated as the proportion of accumulated deficits with a cut-point of ≥0.25 considered frail and <0.25 as nonfrail (25). For example, if a participant had information about 27 items, and 9 deficits were present, an index value of 9/27 = 0.33 would be calculated, and the participant would be considered frail.

### Other Measurements

Data on anthropometry, sociodemographics, lifestyle, and health factors were collected through self-reported or interviewer-administered questionnaires, biochemical analyses, medication inventory, and physical measurements. Height, weight, waist circumference, and hip circumference were measured following standardized protocols as previously described (17). BMI was calculated as kg/m^2^. Physical activity was assessed through PASE.

Marital status was categorized into “married/de facto” and “not married/divorced/separated/widowed/never married/other.” “Age Pension only” referred to those who only received the Age Pension, while “other” referred to those with other sources of income apart from the Age Pension, including veteran pension, repatriation pension, superannuation, private income, business ownership, farm ownership, business partnership, wage, salary and/or other. Country of birth was categorized into “Australia,” “Greece/Italy,” and “other.” Smoking status was categorized into “nonsmoker,” “ex-smoker,” and “current smoker” based on self-reported smoking history. Alcohol consumption was categorized as “nondrinker” for those who had <12 standard (std) drinks in their entire life, “ex-drinker” for those who had <12 std drinks in the past 12 months, “safe drinker” for those who had ≤4 std drinks per day and ≤10 std drinks per week and “harmful drinker” for those who had >4 std drinks per day or >10 std drinks per week (26). The revised Australian Dietary Guideline Index (DGI-2013) is a food-based dietary index developed using a 111-item food frequency questionnaire to investigate compliance with the 2013 Australian Dietary Guidelines (27). The DGI-2013 comprised of 13 components reflecting the criteria of a healthy dietary pattern and each scored out of 10 with an overall possible maximum score of 130, where 0 is considered as low compliance and 10 is considered as better compliance for each component (27). The DGI indicated the overall dietary pattern in the present study, and the adapted criteria to the DGI-2013 have been detailed elsewhere (17).

Prescription and nonprescription medication used daily or almost daily were brought to the baseline clinic visit and recorded. Participants were asked whether they had taken any other medications during the past month. Reported medications were coded using the Iowa Drug Information Service drug code numbers. Iron and/or multivitamin supplement use was categorized into “yes” and “no.” Nonsteroidal anti-inflammatory drug (NSAID) and/or proton pump inhibitor (PPI) use was categorized into “NSAID only,” “PPI only,” “both,” and “neither.” Self-rated health was obtained through response to the question, “compared to other people of your age, how would you rate your health?”, and data was categorized into “very poor/poor/fair” and “good/excellent”. The number of comorbidities was determined by the sum of all conditions that participants reported, including: diabetes, thyroid disease, osteoporosis, Paget’s disease, stroke, Parkinson’s disease, kidney stones, dementia, depression, epilepsy, hypertension, myocardial infarction, angina, congestive cardiac failure, intermittent claudication, chronic obstructive pulmonary disease, liver disease, renal disease, arthritis, gout, and cancer (excluding nonmelanoma skin cancers). Anemia was defined as hemoglobin levels <130 g/L, and those without anemia had hemoglobin levels ≥130 g/L (28). The inflammatory biomarker included was cytokine interleukin-6 (IL-6).

### Statistical Analysis

Statistical analysis was carried out using SPSS software version 25 (IBM Corp., Armonk, NY) (29). Normality tests (histogram, Q–Q plot, and Shapiro-Wilk test) conducted found that most data had a skewed distribution. Descriptive characteristics were expressed as median (interquartile range; IQR) and as number of participants (percentage of participants). Participant characteristics at baseline nutrition according to heme to nonheme iron ratio as a percentage (%) intake were compared through chi-square and median tests. Iron intakes at baseline nutrition and at 3-year follow-up were compared through nonparametric-related samples Wilcoxon signed-rank test.

The associations between iron intakes with frailty status in cross-sectional analyses, and changes in iron intakes with incident frailty in longitudinal analyses, were evaluated through binary logistic regression. For FP frailty, FP robust or FP prefrail versus FP frail were the outcomes of interest. For FI frailty, FI nonfrail versus FI frail were the outcomes of interest. For FP frailty, longitudinal analyses only included participants who were FP robust or FP prefrail at baseline nutrition. Participants remaining FP robust or FP prefrail at baseline nutrition and at 3-year follow-up were compared to those who deteriorated from FP robust or FP prefrail at baseline nutrition to frail at 3-year follow-up. For FI frailty, longitudinal analyses included participants who were FI nonfrail at baseline nutrition. Participants remaining FI nonfrail at baseline nutrition and at 3-year follow-up were compared to those who deteriorated from FI nonfrail at baseline nutrition to FI frail at 3-year follow-up. Iron intakes and changes in iron intakes were evaluated as continuous and categorical variables reported in tables (ie, categorized into 3 tertiles with the low tertile as the reference category). Results are presented as odds ratios (ORs) with 95% confidence intervals (CIs).

Models were adjusted for covariates including sociodemographic and lifestyle factors (age, BMI, country of birth, marital status, age pension, alcohol consumption, smoking status, energy intake, DGI, number of serves of fruits, vegetables, grains, meat/alternatives, dairy/alternatives, and iron and/or multivitamin supplement use) and health (IL-6, NSAID and/or PPI use, self-rated health, and number of comorbidities). The finally adjusted model was conducted with and without the additional adjustment for hemoglobin. The respective baseline iron intakes (total iron, heme iron, nonheme iron, or heme to nonheme iron ratio %) were included as a covariate in longitudinal analyses evaluating changes in the respective iron intake with incident frailty. Physical activity was not included in the model since it is one of the components of frailty. A statistically significant Likelihood Ratio chi-square test and statistically nonsignificant Hosmer–Lemeshow test indicated the goodness of fit of the finally adjusted models. Collinearity diagnostics were conducted, and there was no collinearity in the models with variance inflation factors (VIFs) <2.5 (30), except slightly higher VIFs for energy intake (2.5–2.9) in cross-sectional and longitudinal analyses correlating with food groups, and baseline respective nonheme iron intake (2.6) in longitudinal analyses correlating with changes in nonheme iron intake as expected. There was no evidence that this was an issue for the model as the VIF of independent variables were <2.5 in all analyses.

## Results

A total of 785 men had iron intake and FP frailty data available at baseline collection of nutrition data. Of these, 758 men also had FI frailty data available. The median (IQR) age was 80.0 (77.0–84.0) years and BMI was 27.5 (25.0–30.2) kg/m^2^. Table 1 presents the participant characteristics according to tertiles of heme to nonheme iron ratio % intake. Most characteristics did not differ between the tertiles except for the country of birth, alcohol consumption, energy intake, DGI, number of serves of fruits, vegetables, grains, dairy/alternatives, meat/alternatives, and total iron intake. By definition, heme iron and nonheme iron intakes were also different between the heme to nonheme iron ratio % intake tertiles. The high tertile of heme to nonheme iron ratio % intake had half of the participants born in Australia, a quarter born in Greece or Italy, and a quarter born in other countries were less likely to have been nondrinkers and ex-drinkers, and more likely to have been safe and harmful drinkers. Participants in the high tertile of heme to nonheme iron ratio % intake also had lower energy intake than the medium tertile, lower intake of fruits, vegetables, and grains than the low tertile, and a lower DGI, lower total iron intake, lower dairy/alternatives, and higher meat/alternatives intake than those in the low and medium tertiles.

**Table 1.** Baseline Participant Characteristics According to Heme to Nonheme Iron Ratio as a Percentage Intakes (*n* = 785)

Variables	All	Low Tertile ≤11.97%	Medium Tertile 11.98%–19.39%	High Tertile ≥19.40%	*p* Value*
	*n* (%)	*n* (%)	*n* (%)	*n* (%)	
Age (years)	785	262	262	261	.58
Median (IQR)	80.0 (77.0–84.0)	80.0 (78.0–84.0)	80.0 (77.0–84.0)	81.0 (77.0–84.0)	
BMI (kg/m^2^)	782	261	261	260	.14
Median (IQR)	27.5 (25.0–30.2)	26.9 (24.7–29.8)	27.4 (25.0–30.2)	28.0 (25.2–30.5)	
Waist circumference (cm)	785	262	262	261	.16
Median (IQR)	101.5 (94.1–108.3)	100.1 (93.3–108.1)	101.9 (94.9–108.3)	102.9 (95.5–109.0)	
Hip circumference (cm)	785	262	262	261	.43
Median (IQR)	102.0 (97.5–108.0)	101.2 (96.2–106.1)	102.5 (98.1–109.3)	102.2 (98.0–108.2)	
Marital status	779	258	261	260	.69
Married/de facto	592 (76.0)	195 (75.6)	203 (77.8)	194 (74.6)	
Not married/divorced/separated/widowed/never married/other	187 (24.0)	63 (24.4)	58 (22.2)	66 (25.4)	
Source of income	783	262	260	261	.64
Age pension only	314 (40.1)	105 (40.1)	99 (38.1)	110 (42.1)	
Other	469 (59.9)	157 (59.9)	161 (61.9)	151 (57.9)	
Country of birth	785	262	262	261	.008
Australia	412 (52.5)	120 (45.8)	161 (61.5)	131 (50.2)	
Greece/Italy	186 (23.7)	72 (27.5)	51 (19.5)	63 (24.1)	
Other	187 (23.8)	70 (26.7)	50 (19.1)	67 (25.7)	
Cigarette smoking status	778	261	258	259	.88
Nonsmoker	316 (40.6)	105 (40.2)	110 (42.6)	101 (39.0)	
Ex-smoker	434 (55.8)	148 (56.7)	139 (53.9)	147 (56.8)	
Current smoker	28 (3.6)	8 (3.1)	9 (3.5)	11 (4.2)	
Alcohol consumption	784	262	262	260	<.001
Nondrinker (<12 std drinks in entire life)	73 (9.3)	32 (12.2)	23 (8.8)	18 (6.9)	
Ex-drinker (<12 std drinks in past 12 months)	105 (13.4)	50 (19.1)	36 (13.7)	19 (7.3)	
Safe drinker (≤4 std drinks per day and ≤10 std drinks per week)	339 (43.2)	114 (43.5)	103 (39.3)	122 (46.9)	
Harmful drinker (>4 std drinks per day or >10 std drinks per week)	267 (34.1)	66 (25.2)	100 (38.2)	101 (38.8)	
Iron and/or multivitamin supplement use	785	262	262	261	.098
Yes	19 (2.4)	8 (3.1)	2 (0.8)	9 (3.4)	
No	766 (97.6)	254 (96.9)	260 (99.2)	252 (96.6)	
NSAID and/or PPI use					.21
NSAID only	221 (28.2)	80 (30.5)	70 (26.7)	71 (27.2)	
PPI only	138 (17.6)	44 (16.8)	38 (14.5)	56 (21.5)	
Both	88 (11.2)	33 (12.6)	33 (12.6)	22 (8.4)	
Neither	338 (43.1)	105 (40.1)	121 (46.2)	112 (42.9)	
Anemia	759	255	253	251	.82
Yes	132 (17.4)	47 (18.4)	44 (17.4)	41 (16.3)	
No	627 (82.6)	208 (81.6)	209 (82.6)	210 (83.7)	
Hemoglobin (g/L)	759	255	253	251	.24
Median (IQR)	143.0 (134.0–152.0)	142.0 (133.0–151.0)	143.0 (134.0–151.5)	144.0 (134.0–153.0)	
Interleukin-6 (pg/mL)	716	233	245	238	.26
Median (IQR)	2.5 (1.3–4.8)	2.3 (1.2–4.1)	2.5 (1.3–4.9)	2.9 (1.4–5.1)	
Self-rated health	784	262	261	261	.53
Very poor/poor/fair	201 (25.6)	66 (25.2)	62 (23.8)	73 (28.0)	
Good/excellent	583 (74.4)	196 (74.8)	199 (76.2)	188 (72.0)	
Number of comorbidities	784	262	261	261	.57
Median (IQR)	2.0 (1.0–3.0)	2.0 (1.0–3.0)	2.0 (1.0–4.0)	2.0 (1.0–3.0)	
PASE	784	262	261	261	.49
Median (IQR)	121.0 (77.1–161.7)	126.9 (76.4–158.2)	121.0 (78.8–161.7)	116.7 (70.9–165.0)	
Energy intake (kJ)	785	262	262	261	.038^a^
Median (IQR)	8 824.6 (7 311.1–10 515.4)	8 855.2 (7 359.2–10 497.9)	9 137.7 (7 626.2–10 766.6)^a^	8 527.2 (6 972.9–10 200.1)^a^	
DGI	785	262	262	261	<.001^a,b^
Median (IQR)	93.7 (87.5–101.1)	95.7 (89.4–102.6)^a^	95.0 (88.2–101.8)^b^	90.0 (84.3–97.8)^a,b^	
Number of fruit serves	785	262	262	261	.008^a^
Median (IQR)	1.8 (1.1–2.7)	2.0 (1.3–3.0)^a^	1.8 (1.1–2.7)	1.6 (0.9–2.3)^a^	
Number of vegetable serves	785	262	262	261	.002^a^
Median (IQR)	3.5 (2.4–4.9)	3.8 (2.5–5.5)^a^	3.5 (2.5–4.7)	3.1 (2.3–4.5)^a^	
Number of grain serves	785	262	262	261	<.001^a^ .003^b^
Median (IQR)	5.0 (3.7–6.4)	5.5 (4.2–7.1)^a,b^	4.9 (3.7–6.2)^b^	4.4 (3.3–6.0)^a^	
Number of meat/alternative serves	785	262	262	261	<.001^a,b,c^
Median (IQR)	2.9 (2.2–3.7)	2.4 (1.7–3.2)^a,b^	2.8 (2.3–3.6)^a,c^	3.2 (2.7–4.1)^b,c^	
Number of dairy/alternative serves	785	262	262	261	.001^a^ <.001^b^
Median (IQR)	1.7 (1.1–2.4)	1.9 (1.2–2.5)^a^	1.9 (1.2–2.7)^b^	1.5 (0.9–2.2)^a,b^	
Total iron (mg/d)	785	262	262	261	<.001^a^ .038^b^
Median (IQR)	12.9 (10.5–16.0)	14.1 (11.0–18.1)^a^	13.0 (10.9–15.7)^b^	11.9 (9.8–14.6)^a,b^	
Heme iron (mg/d)	785	262	262	261	<.001^a,b,c^
Median (IQR)	1.7 (1.2–2.4)	1.0 (0.7–1.4)^a,b^	1.8 (1.4–2.2)^a,c^	2.5 (2.0–3.1)^b,c^	
Nonheme iron (mg/d)	785	262	262	261	.001^a^ <.001^b,c^
Median (IQR)	11.0 (8.8–13.9)	12.9 (10.2–16.4)^a,b^	11.3 (9.4–13.5)^a,c^	9.4 (7.7–11.5)^b,c^	

*Notes:* BMI = body mass index; DGI = Australian Dietary Guideline Index; IQR = interquartile range; NSAID = nonsteroidal anti-inflammatory drug; PASE = Physical Activity Scale for the Elderly; PPI = proton pump inhibitor; std = standard.

*p Values were obtained using the median test to compare all tertile groups for differences in median values of continuous variables. Differences between groups are denoted by each letter a, b, or c. *p* Values were obtained using the chi-square test to compare all tertile groups for differences in proportions of participants in categories for categorical variables.

The prevalence of FP frailty was 8.3% (*n* = 65) and of FI frailty was 31% (*n* = 234) at baseline nutrition. Cross-sectional analyses evaluating associations between iron intakes and FP frailty status are shown in Table 2. Compared to the low tertile (≤11.33 mg/d) of total iron intakes, those in the medium (11.34–14.93 mg/d) and high tertiles (≥14.94 mg/d) had a lower prevalence of FP frailty in unadjusted analyses (OR: 0.48 [95% CI: 0.26, 0.89, *p* = .019] and OR: 0.42 [95% CI: 0.22, 0.80, *p* = .008], respectively). However, there were no associations in the fully adjusted model. When analyzed as a continuous variable, higher total iron intake (each 1 mg increment) was not associated with FP frailty status in unadjusted and fully adjusted analyses. Compared to the low tertile (≤1.38 mg/d) of heme iron intakes, participants in the high tertile (≥2.14 mg/d) had a lower prevalence of FP frailty in unadjusted analyses (OR: 0.45 [95% CI: 0.24, 0.86, *p* = .016]), but there was no association in the fully adjusted model. Higher heme iron intake (each 1 mg increment) was also not associated with FP frailty status in unadjusted and fully adjusted analyses. Compared to the low tertile (≤9.55 mg/d) of nonheme iron intakes, participants in the medium (9.56–12.81 mg/d) and high tertiles (≥12.82 mg/d) had lower prevalence of FP frailty in unadjusted analyses (OR: 0.42 [95% CI: 0.22, 0.80, *p* = .008] and OR: 0.48 [95% CI: 0.26, 0.89, *p* = .020], respectively). Higher nonheme iron intakes (each 1 mg increment) was also associated with lower prevalence of FP frailty in unadjusted analyses (OR: 0.90 [95% CI: 0.84, 0.97, *p* = .006]). However, there were no associations between nonheme iron intakes and FP frailty status in the fully adjusted model. There were no cross-sectional associations between FI frailty and all dietary iron intakes in unadjusted and adjusted analyses (Supplementary Table 1).

**Table 2.** Cross-sectional Associations Between Dietary Iron Intakes and FP Frailty Status (*n* = 785)

Iron Intake	Low Tertile (Reference Category)	Medium Tertile	High Tertile	As Continuous Variable
Total iron*				
Model 1	1	0.48 (0.26, 0.89) *p* = .019	0.42 (0.22,0.80) *p* = .008	0.98 (0.93, 1.02) *p* = .29
Model 2	1	0.70 (0.33, 1.48) *p* = .35	0.48 (0.19, 1.20) *p* = .12	0.98 (0.94, 1.02) *p* = .22
Model 3				
Without hemoglobin	1	0.77 (0.32, 1.89) *p* = .57	0.47 (0.16, 1.43) *p* = .19	1.00 (0.96, 1.03) *p* = .87
With hemoglobin	1	0.75 (0.30, 1.91) *p* = .55	0.52 (0.17, 1.62) *p* = .26	1.00 (0.96, 1.03) *p* = .77
Heme iron^†^				
Model 1	1	0.58 (0.32, 1.06) *p* = .077	0.45 (0.24, 0.86) *p* = .016	0.77 (0.57, 1.03) *p* = .074
Model 2	1	0.78 (0.38, 1.61) *p* = .50	0.59 (0.24, 1.42) *p* = .24	0.93 (0.62, 1.40) *p* = .72
Model 3				
Without hemoglobin	1	0.74 (0.29, 1.78) *p* = .51	0.61 (0.21, 1.78) *p* = .36	1.00 (0.61, 1.63) *p* = .99
With hemoglobin	1	0.72 (0.28, 1.85) *p* = .50	0.65 (0.21, 2.07) *p* = .47	1.00 (0.59, 1.69) *p* = 1.00
Nonheme iron^‡^				
Model 1	1	0.42 (0.22, 0.80) *p* = .008	0.48 (0.26, 0.89) *p* = .020	0.90 (0.84, 0.97) *p* = .006
Model 2	1	0.63 (0.29, 1.35) *p* = .23	0.55 (0.22, 1.41) *p* = .21	0.93 (0.83, 1.03) *p* = .15
Model 3				
Without hemoglobin	1	0.60 (0.23, 1.56) *p* = .30	0.55 (0.18, 1.68) *p* = .30	0.95 (0.83, 1.08) *p* = .41
With hemoglobin	1	0.70 (0.26, 1.85) *p* = .47	0.65 (0.21, 2.05) *p* = .47	0.96 (0.83, 1.09) *p* = .50
Heme to nonheme iron ratio %^§^				
Model 1	1	1.48 (0.76, 2.87) *p* = .25	1.70 (0.89, 3.25) *p* = .11	1.01 (0.99, 1.04) *p* = .41
Model 2	1	1.90 (0.83, 4.34) *p* = .13	2.14 (0.90, 5.11) *p* = .086	1.01 (0.98, 1.05) *p* = .44
Model 3				
Without hemoglobin	1	2.08 (0.77, 5.61) *p* = .15	1.76 (0.61, 5.14) *p* = .30	1.02 (0.98, 1.06) *p* = .42
With hemoglobin	1	1.97 (0.73, 5.34) *p* = .18	1.88 (0.63, 5.63) *p* = .26	1.02 (0.97, 1.06) *p* = .49

*Notes:* BMI = body mass index; FP = frailty phenotype; IL-6 = interleukin-6; IQR = interquartile range; NSAID = nonsteroidal anti-inflammatory drug; PPI = proton pump inhibitor. Model 1 unadjusted (*n* = 785 for total, 65 frail); Model 2 adjusted by sociodemographic and lifestyle factors (age, BMI, country of birth, marital status, age pension, alcohol consumption, smoking status, energy intake, Australian Dietary Guideline Index, fruits, vegetables, grains, meat/alternatives, dairy/alternatives iron, and/or multivitamin supplement use; *n* = 768 for total, 63 frail); Model 3 adjusted by Model 2 plus health (IL-6, NSAID and/or PPI use, self-rated health, and number of comorbidities) without hemoglobin (*n* = 699 for total, 51 frail) and with hemoglobin (*n* = 694 for total, 50 frail)

*Low tertile ≤11.33 mg/d, *n* = 262 with median (IQR) 9.52 (8.16, 10.54); medium tertile 11.34–14.93 mg/d, *n* = 262 with median (IQR) 12.94 (12.11, 13.93); high tertile ≥14.94 mg/d, *n* = 261 with median (IQR) 17.75 (15.98, 20.18).

^†^Low tertile ≤1.38 mg/d, *n* = 262 with median (IQR) 1.00 (0.72, 1.19); medium tertile 1.39–2.13 mg/d, *n* = 262 with median (IQR) 1.74 (1.55, 1.93); high tertile ≥2.14 mg/d, *n* = 261 with median (IQR) 2.70 (2.40, 3.20).

^‡^Low tertile ≤9.55 mg/d, *n* = 262 with median (IQR) 7.99 (6.63, 8.79); medium tertile 9.56–12.81 mg/d, *n* = 262 with median (IQR) 11.03 (10.39, 11.87); high tertile ≥12.82 mg/d, *n* = 261 with median (IQR) 15.46 (13.90, 17.78).

^§^Low tertile ≤11.95 %/d, *n* = 262 with median (IQR) 8.61 (6.20, 10.27); medium tertile 11.96–19.39 %/d, *n* = 262 with median (IQR) 15.25 (13.83, 17.46); high tertile ≥19.40 %/d, *n* = 261 with median (IQR) 25.49 (22.31, 31.91).

Of the 720 men who were FP robust or FP prefrail at baseline nutrition assessment, 563 had data available at 3-year follow-up. Data was unavailable for 157 men at 3-year follow-up due to: 64 men did not complete any assessments (29 were too ill or unable, 12 moved out of the area, 5 were not interested, 5 felt they did enough for the study, 5 provided no reasons, 3 were busy, 2 declined due to family concerns, 1 had limited English literacy, 1 was unable to be contacted, and 1 withdrew completely from the project), 85 men did not agree to the nutrition component and 8 men who had dietary data did not have FP frailty data. Of the 524 men who were FI nonfrail at baseline nutrition assessment, 432 men had data available at 3-year follow-up. Data was unavailable for 92 men at 3-year follow-up due to: 41 men did not complete any assessments (12 were too ill or unable, 8 moved out of the area, 4 were not interested, 5 felt they did enough for the study, 5 provided no reasons, 3 were busy, 2 declined due to family concerns, 1 had limited English literacy, and 1 was unable to be contacted) and 51 men did not agree to the nutrition component.

Table 3 shows the median daily iron intakes, changes in iron intake and top sources of iron at baseline nutrition and 3-year follow-up. The top sources of total iron were identical and similar to the top sources of nonheme iron but not heme iron, at baseline nutrition and 3-year follow-up, respectively. “Breakfast cereal, whole wheat, biscuit, added vitamins B1, B2, B3 & folate, and Fe & Zn,” “beef, rump steak, lean, and grilled,” and “breakfast cereal, flakes of corn, added vitamins B1, B2, B3 & folate, and Fe” remained in the top sources of both total iron and nonheme iron, however, “chocolate, dark, and high cocoa solids” and “muesli, commercial, toasted, and unfortified” were replaced by “bread, from wholemeal flour” and “bread, from wholemeal flour, grain & seeds” at 3-year follow-up. “Beef, rump steak, lean, and grilled,” “hamburger patty, frozen, and grilled,” and “lamb, loin chop, lean, and grilled” remained in the top sources of heme iron; however, “lamb, cooked, and not further specified” and “chicken, grilled/BBQ, and not further specified” were replaced by “beef, rump steak, lean, and baked/roasted” and “beef, stew/casserole, tomato sauce, and vegetables including potato” at 3-year follow-up. Median daily intakes of total iron, heme iron, and nonheme iron decreased at 3-year follow-up compared to baseline nutrition (all *p* < .001), but heme to nonheme iron ratio % did not change. The median (IQR) changes in total iron, heme iron, and nonheme iron intakes were −1.04 mg/d (−3.68,1.69), −0.11 mg/d (−0.83,0.40), and −0.62 mg/d (−2.90,1.45), respectively.

**Table 3.** Median Daily Dietary Iron Intakes, Median Changes in Dietary Iron Intakes and Top Sources of Dietary Iron at Baseline Nutrition and 3-Year Follow-up (*n* = 563)

Iron Intake	Baseline		Three-year		Change	*p* Value*
	Median (IQR)	Top Sources	Median (IQR)	Top Sources	Median (IQR)	
Total iron (mg/d)	13.10 (10.84, 15.99)	i. Breakfast cereal, whole wheat, biscuit, added vitamins B1, B2, B3, & folate, Fe & Zn ii. Beef, rump steak, lean, grilled iii. Chocolate, dark, high cocoa solids iv. Muesli, commercial, toasted, unfortified v. Breakfast cereal, flakes of corn, added vitamins B1, B2, B3, & folate, Fe	12.13 (9.98, 15.04)	i. Breakfast cereal, whole wheat, biscuit, added vitamins B1, B2, B3, & folate, Fe & Zn ii. Beef, rump steak, lean, grilled iii. Breakfast cereal, flakes of corn, added vitamins B1, B2, B3, & folate, Fe iv. Bread, from wholemeal flour v. Bread, from wholemeal flour, grain & seeds	−1.04 (−3.68, 1.69)	<.001
Heme iron (mg/d)	1.75 (1.21, 2.41)	i. Beef, rump steak, lean, grilled ii. Hamburger patty, frozen, grilled iii. Lamb, cooked, not further specified iv. Lamb, loin chop, lean, grilled v. Chicken, grilled/BBQ, not further specified	1.54 (1.07, 2.10)	i. Beef, rump steak, lean, grilled ii. Hamburger patty, frozen, grilled iii. Lamb, loin chop, lean, grilled iv. Beef, rump steak, lean, baked/roasted v. Beef, stew/casserole, tomato sauce, vegetables including potato	−0.11 (−0.83, 0.40)	<.001
Nonheme iron (mg/d)	11.28 (8.93, 13.94)	i. Breakfast cereal, whole wheat, biscuit, added vitamins B1, B2, B3, & folate, Fe & Zn ii. Beef, rump steak, lean, grilled iii. Chocolate, dark, high cocoa solids iv. Muesli, commercial, toasted, unfortified v. Breakfast cereal, flakes of corn, added vitamins B1, B2, B3, & folate, Fe	10.40 (8.59, 13.09)	i. Breakfast cereal, whole wheat, biscuit, added vitamins B1, B2, B3, & folate, Fe & Zn ii. Breakfast cereal, flakes of corn, added vitamins B1, B2, B3, & folate, Fe iii. Beef, rump steak, lean, grilled iv. Bread, from wholemeal flour v. Bread, from wholemeal flour, grain & seeds	−0.62 (−2.90, 1.45)	<.001
Heme to nonheme iron ratio (%/d)	14.93 (10.22, 22.23)	―	14.93 (10.11, 20.53)	―	−0.22 (−6.06, 4.43)	.12

*Note:* IQR = interquartile range.

^1^ *p* Values were obtained using the related samples Wilcoxon signed-rank test to compare the differences between median intakes at baseline nutrition and at 3-year follow-up.

Of the 563 men who were FP robust or FP prefrail at baseline nutrition and seen at 3-year follow-up, the following transitions in FP frailty status occurred: 22.0% remained robust (*n* = 124), 27.4% deteriorated from robust to prefrail (*n* = 154), 2.7% deteriorated from robust to frail (*n* = 15), 3.9% improved from prefrail to robust (*n* = 22), 31.4% remained prefrail (*n* = 177), and 12.6% deteriorated from prefrail to frail (*n* = 71). Of the 432 men who were FI nonfrail at baseline nutrition and seen at 3-year follow-up, the following transitions in FI frailty status occurred: 45.8% remained nonfrail (*n* = 124) and 54.2% deteriorated from nonfrail to frail (*n* = 234). The median daily iron intakes and changes in iron intakes according to transitions in FP and FI frailty status are available in Supplementary Tables 2 and 3, respectively.

The incidence of FP frailty was 15.3% (*n* = 86) and of FI frailty was 54.2% (*n* = 234) at 3-year follow-up. Longitudinal analyses evaluating associations between changes in iron intakes and incident FP frailty are presented in Table 4. Compared to decreased total iron intake (low tertile ≤−2.62 mg/d), maintaining total iron intakes (medium tertile −2.61–0.81 mg/d) was not associated with incident FP frailty in unadjusted analyses but was associated with reduced risks of incident FP frailty in both fully adjusted models without hemoglobin (OR: 0.45 [95% CI: 0.23, 0.89, *p* = .021]) and with hemoglobin (OR: 0.47 [95% CI: 0.24, 0.93, *p* = .031]). Increasing total iron intake (high tertile ≥0.82 mg/d) was not associated with incident FP frailty in unadjusted analyses and in the fully adjusted model without hemoglobin but was associated with reduced risks of incident FP frailty in the fully adjusted model without hemoglobin (OR 0.46 [95% CI: 0.22, 0.96, *p* = .038]) and with hemoglobin (OR 0.48 [95% CI: 0.23, 0.99, *p* = .048]). As a continuous variable, changes in total iron intake (each 1 mg increment) were not associated with incident FP frailty in unadjusted and fully adjusted analyses.

**Table 4.** Longitudinal Associations Between Changes in Dietary Iron Intakes and Incident FP Frailty (*n* = 563)

Iron Intake	Low Tertile (Reference Category)	Medium Tertile	High Tertile	As Continuous Variable
Total iron*				
Model 1	1	0.65 (0.37, 1.13) *p* = .13	0.65 (0.37, 1.14) *p* = .13	1.00 (0.97, 1.03) *p* = 1.00
Model 2	1	0.51 (0.27, 0.96) *p* = .036	0.70 (0.37, 1.31) *p* = .26	1.01 (0.98, 1.04) *p* = .64
Model 3				
Without hemoglobin	1	0.45 (0.23, 0.89) *p* = .021	0.46 (0.22, 0.96) *p* = .038	1.00 (0.97, 1.04) *p* = .86
With hemoglobin	1	0.47 (0.24, 0.93) *p* = .031	0.48 (0.23, 0.99) *p* = .048	1.00 (0.97, 1.04) *p* = .82
Heme iron^†^				
Model 1	1	1.00 (0.58, 1.72) *p* = 1.00	0.75 (0.42, 1.33) *p* = .32	0.93 (0.75, 1.15) *p* = .49
Model 2	1	1.02 (0.56, 1.88) *p* = .94	0.85 (0.44, 1.61) *p* = .61	0.98 (0.77, 1.26) *p* = .88
Model 3				
Without hemoglobin	1	0.97 (0.46, 2.04) *p* = .93	0.73 (0.34, 1.58) *p* = .42	0.83 (0.59, 1.16) *p* = .28
With hemoglobin	1	0.94 (0.45, 1.99) *p* = .88	0.70 (0.32, 1.52) *p* = .37	0.82 (0.59, 1.15) *p* = .26
Nonheme iron^‡^				
Model 1	1	0.56 (0.32, 0.97) *p* = .040	0.54 (0.31, 0.94) *p* = .029	0.94 (0.89, 0.99) *p* = .019
Model 2	1	0.45 (0.24, 0.85) *p* = .013	0.52 (0.27, 0.98), *p* = .042	0.93 (0.87, 0.99) *p* = .023
Model 3				
Without hemoglobin	1	0.51 (0.26, 1.01) *p* = .054	0.40 (0.19, 0.85) *p* = .017	0.89 (0.82, 0.98) *p* = .015
With hemoglobin	1	0.53 (0.27, 1.06) *p* = .071	0.41 (0.20, 0.88) *p* = .022	0.89 (0.82, 0.98) *p* = .017
Heme to nonheme iron ratio %^§^				
Model 1	1	0.78 (0.45,1.37) *p* = .39	0.86 (0.49, 1.49) *p* = .59	1.01 (0.99, 1.03) *p* = .53
Model 2	1	0.80 (0.43, 1.50) *p* = .49	0.87 (0.47, 1.61) *p* = .66	1.01 (0.99, 1.04) *p* = .30
Model 3				
Without hemoglobin	1	0.58 (0.27, 1.22) *p* = .15	0.64 (0.30, 1.35) *p* = .24	1.01 (0.97, 1.04) *p* = .79
With hemoglobin	1	0.55 (0.26, 1.18) *p* = .12	0.60 (0.28, 1.29) *p* = .19	1.00 (0.97, 1.04) *p* = .85

*Notes*: BMI = body mass index; FP = frailty phenotype; IL-6 = interleukin-6; IQR = interquartile range; NSAID = nonsteroidal anti-inflammatory drug; PPI = proton pump inhibitor. Model 1 unadjusted (*n* = 563 for total, 86 frail); Model 2 adjusted by sociodemographic and lifestyle factors (age, BMI, country of birth, marital status, age pension, alcohol consumption, smoking status, energy intake, Australian Dietary Guideline Index, number of serves of fruits, vegetables, grains, dairy/alternatives and meat/alternatives, iron, and/or multivitamin supplement use; *n* = 553 for total, 85 frail); Model 3 adjusted by Model 2 plus health and respective baseline iron intake (IL-6, NSAID, and/or PPI use, self-rated health, number of comorbidities and respective baseline iron intake) without hemoglobin (*n* = 506 for total, 77 frail) and with hemoglobin (*n* = 502 for total, 77 frail).

*Low tertile ≤−2.62 mg/d, *n* = 188 with median (IQR) −4.93 (−6.94, −3.63); medium tertile −2.61–0.81 mg/d, *n* = 188 with median (IQR) −1.04 (−1.74, 0.00); high tertile ≥0.82 mg/d, *n* = 187 with median (IQR) 2.94 (1.69, 5.00).

^†^Low tertile ≤−0.51 mg/d, *n* = 188 with median (IQR) −1.11 (−1.63, −0.83); medium tertile −0.50–0.22 mg/d, *n* = 188 with median (IQR) −0.11 (−0.34, 0.04); high tertile ≥0.23 mg/d, *n* = 187 with median (IQR) 0.64 (0.40, 1.13).

^‡^Low tertile ≤−2.10 mg/d, *n* = 188 with median (IQR) −4.25 (−5.92, −2.87); medium tertile −2.09–0.79 mg/d, *n* = 188 with median (IQR) −0.62 (−1.28, 0.23); high tertile ≥0.80 mg/d, *n* = 187 with median (IQR) 2.50 (1.45, 4.49).

^§^Low tertile ≤−3.95 %/d, *n* = 188 with median (IQR) −9.23 (−14.77, −6.04); medium tertile −3.94–2.88 %/d, *n* = 188 with median (IQR) −0.20 (−1.79, 1.34); high tertile ≥2.89 %/d, *n* = 187 with median (IQR) 7.22 (4.43, 11.45).

Compared to participants who decreased their nonheme iron intake (low tertile ≤−2.10 mg/d), those who maintained or increased (medium tertile −2.09–0.79 mg/d and high tertile ≥0.80 mg/d) had reduced risks of incident FP frailty in unadjusted analyses (OR 0.56 [95% CI: 0.32, 0.97, *p* = .040] and OR 0.54 [95% CI: 0.31, 0.94, *p* = .029], respectively). Maintaining nonheme iron intake (medium tertile −2.09–0.79 mg/d) was not associated with incident FP frailty in both fully adjusted models without and with hemoglobin. Those who increased their nonheme iron intake (high tertile ≥0.80 mg/d) remained at reduced risk of incident FP frailty in the fully adjusted model without hemoglobin (OR 0.40 [95% CI: 0.19, 0.85, *p* = .017]) and with hemoglobin (OR 0.41 [95% CI: 0.20, 0.88, *p* = .022]). As a continuous variable, changes in nonheme iron intake (each 1 mg increment) was associated with reduced risks of incident FP frailty in unadjusted analyses (OR 0.94 [95% CI: 0.89, 0.99, *p* = .019]), and in both fully adjusted models without hemoglobin (OR 0.89 [95% CI: 0.82, 0.98, *p* = .015]) and with hemoglobin (OR 0.89 [95% CI: 0.82, 0.98, *p* = .017]). There were no longitudinal associations between FI frailty and all dietary iron intakes in unadjusted and adjusted analyses (Supplementary Table 4).

## Discussion

To the best of the authors’ knowledge, this study is the first study to examine cross-sectional associations between dietary iron intakes (total iron, heme iron, nonheme iron, and heme to nonheme iron ratio) with frailty status and longitudinal associations between changes in these dietary iron intakes with incident frailty in older men. We found no cross-sectional associations between iron intakes and frailty status determined through both FP and FI criteria. Our longitudinal analyses of more than 3 years revealed that compared to substantial decline, maintaining or increases in total iron intakes was associated with reduced risks of incident FP frailty in older men without and with hemoglobin adjustment. Increases and changes in nonheme iron intakes without and with hemoglobin adjustment were also associated with reduced risks of FP incident frailty in older men. We found no longitudinal associations between iron intakes and incident FI frailty. The top sources of total iron and nonheme iron were also similar at each timepoint, which further demonstrates that nonheme iron rather than heme iron is a larger contributor to total iron intake.

The evaluation of associations between dietary iron intakes rather than iron status biomarkers with frailty allows for more meaningful applications. Previous research has shown that associations between dietary iron intakes and iron status biomarkers in older men had been inconsistent (4,31–34), with the lack of association possibly due to the presence of chronic low-grade inflammation in aging known as “inflammaging” (35). Maintenance or increases in total iron intakes, as well as increases or changes in nonheme iron intakes can be practically recommended and implemented. Increases of ≥0.82 mg/d of total iron and ≥0.80 mg/d of nonheme iron can be achieved through additional amounts of various food sources such as quarter cup muesli or iron-fortified breakfast cereal, half slice iron-fortified bread, quarter cup cooked spinach, half cup cooked green beans or peas, half cup baked beans, half-square tofu, 6 dried apricot halves, 1 cup strawberries or 2 tablespoons of cashews, pine nuts, pistachios, or almonds (36).

A previous cross-sectional study found no differences in total iron intakes between older men who were FP prefrail or robust (37). Although the study only investigated FP prefrailty and involved a Japanese population where the food supply likely contained different heme iron content from the various fish species consumed (38), these findings are in agreement with our cross-sectional analysis findings that involved both FP and FI frailty criteria. However, in our longitudinal analyses we found that changes in total iron and nonheme iron intakes were associated with reduced risks of incident FP frailty but was not associated with incident FI frailty.

The mechanisms by which dietary iron intakes influence the development of frailty are unknown (39). In the present study, we found that maintaining or increases in total iron intakes, and increases or changes in nonheme iron intakes were associated with reduced risks of incident FP frailty without adjusting for hemoglobin. Iron deficiency anemia causing hypoxia is a potential biologically plausible pathway for decline in muscle oxygenation and the deleterious effects on muscle strength, physical and cognitive performance (39,40). However, anemia which was previously associated with increased risks of FP frailty in the CHAMP cohort, could also indirectly reflect inflammaging and the association between anemia and biomarkers of frailty (11). When hemoglobin was accounted for, the associations remained. Another mechanism for the impact of dietary iron intakes on frailty is that iron deficiency without anemia can affect iron-containing compounds other than hemoglobin involved in energy production (myoglobin, oxidative enzymes, and respiratory chain proteins) (16,40–43). This could affect energy production and negatively impact on frailty as discussed later.

In in vitro and animal studies, iron deficiency has been shown to influence muscle energy metabolism with shifts from aerobic to anaerobic energy metabolism, to limit processes with high iron expenditure, and reduce aerobic capacity (16). Iron deficiency, irrespective of anemia has been associated with reduced aerobic capacity in patients with chronic obstructive pulmonary disease, and in endurance athletes whereby iron repletion improved systemic iron status biomarkers and physical performance (16,41,44). Similarly, in patients with heart failure and iron deficiency with or without anemia, depletion of iron stores indicated by low ferritin was associated with muscle weakness, and iron repletion resulted in improvement in walking distance (16,42). A prospective cohort study in older hospitalized patients found that iron deficiency is an independent modifiable risk factor of fatigue, which could lead to lower functional performance and muscle disuse, subsequent decline in muscle strength and weakness (43). The study also found that iron supplementation improved muscle strength (43). Iron deficiency has therefore been highlighted as an emerging therapeutic target in chronic diseases accompanied by marked muscle dysfunction (16), which could similarly be applied to frailty. The different longitudinal findings between changes in iron intakes with incident FP and FI frailty, could be explained by the different components in the criteria where muscle strength, slowness, exhaustion, and low activity is directly included in the FP (20), while the FI indirectly assesses these through deficits of disability and symptoms (24,45).

In a longitudinal study involving adults aged 50–79 years in Australia, energy-adjusted total iron intake was positively associated with the change in appendicular lean mass (46). Recent research found that low iron availability in muscle cells in in vitro experiments, hence deprivation of iron-containing proteins, attenuated muscle protein synthesis, including skeletal muscle (47). This could be one of the mechanisms on how dietary iron intakes influence the development of frailty, as the frailty component of weight loss is a proxy for the loss of muscle mass in the FP criteria (20,48). Thus, other frailty assessment tools that do not encompass muscle mass, such as the FI used for subanalyses in the current study, failed to detect such associations (24,45).

The different associations between changes in heme iron and nonheme iron intakes with incident FP frailty in the present study could be explained by the observation that the top sources of total iron were similar to the top sources of nonheme iron rather than heme iron at each timepoint. Despite the differences in the bioavailability of the different forms of iron, nonheme iron has been shown to contribute to a greater percentage of total bioavailable iron (49). Furthermore, research has shown there is greater relative adaption of enhanced bioavailability of nonheme iron with increased absorption in the state of iron deficiency (49). As the top sources of total iron and nonheme iron included iron-fortified foods, future research should also evaluate the associations between supplementary iron intakes and frailty.

It is important to highlight that our longitudinal analyses examined changes in dietary iron intakes, where compared to substantial decline, maintaining or increases in total iron, and increases or changes in nonheme iron intakes were associated with reduced risks of incident FP frailty. This does not reflect iron overload, whereby the form of hereditary hemeochromatosis has been associated with increased risks of FP frailty (50), and iron overload has also been associated with reduced life expectancy (51). Median dietary iron intakes and median changes in iron intakes in the current study were also well below the upper limit of 45 mg per day for adults (52). A recent umbrella review showed that dietary total iron intake was associated with reduced risks whilst heme iron intake was associated with increased risks of multiple health outcomes (53), indicating the potential survival benefit of total dietary iron and nonheme iron intakes.

There are a number of study limitations. Firstly, selection bias due to loss to follow-up could occur when those who are less healthy become frail and drop out of the study or died prior to follow-up assessment. We did not have data for those lost to follow-up, so we are unable to investigate whether there were differences in exposure and outcome between participants and nonparticipants. The period of follow-up of more than 3 years was relatively short. Nevertheless, in our study, we showed that total dietary iron, heme iron, and nonheme iron intakes changed from baseline nutrition to 3-year follow-up. Heme to nonheme iron ratio % intake did not change. There is also interindividual variation in the bioavailability of iron due to different meal compositions with potential enhancers and inhibitors present that were not accounted for (49). We did not account for supplementary iron intakes as detailed data on supplements were unavailable but adjusted for iron and/or multivitamin supplement use as a categorical covariate. Thus, our study conclusions are based on dietary iron intakes and are not extrapolated to supplementary iron intakes which requires further research. As there was a small number of participants who deteriorated from FP robust to FP frail, we used a dichotomized outcome variable of FP robust or FP prefrail versus FP frail which did not differentiate between FP robust and FP prefrail. There was also a small number of participants who improved from FP prefrail to FP robust which limited exploration of associations between iron intakes and improvements in FP frailty status. Our study was limited to community-dwelling men, and the results may not apply to older women.

The strength of our study is that we explored the relationships of both cross-sectional associations between iron intakes with frailty status and longitudinal associations between changes in iron intakes with incident frailty overtime. We also used 2 measures of frailty, FP, and FI criteria. Examining dietary iron intakes as exposure rather than iron status biomarkers that could be volatile in inflammaging also allows for application as dietary recommendations and practical implementation by individuals to reduce the onset of frailty. We used a validated dietitian-administered diet history questionnaire, which has been indicated for older adults due to the nonreliance on short-term memory and low respondent burden (10). We also adjusted for covariates including the respective baseline iron intake in longitudinal analyses, NSAID and/or PPI use that could cause gastrointestinal bleeding, enteropathy and affect iron absorption, energy intake that would also account for under or overreporting, and the DGI and individual food groups for the overall dietary pattern consumed. We also had dietary exposure data available at both timepoints. A further strength of CHAMP is that it includes a large and representative group of older Australian men (9).

Future research is required to consolidate the possible mechanisms and conduct randomized controlled trials examining the effects of interventions involving total dietary iron, dietary nonheme iron, and supplementary iron intakes on preventing frailty as well as improvements to frailty status. Subgroups consuming only nonheme iron sources should also be included. Consumption of nonheme iron sources to increase nonheme iron, maintain and increase total iron intakes could also allow for better alignment with recommendations for more plant-based dietary patterns to support environmental sustainability, provide other health benefits and allow for sustainable nutrition (54).

In conclusion, we have shown that maintaining or increases in total dietary iron, and increases or changes in dietary nonheme iron intakes appear to reduce the onset of FP frailty in older men. Future research is required to confirm the mechanisms in which dietary iron intake influences the development of frailty and should include those consuming plant-based dietary patterns with nonheme iron intake as the sole contributor to total iron intake.

## Supplementary Material

glac077_suppl_Supplementary_Tables
