# Supplementary material for: Changes in Dietary Total and Nonheme Iron Intake Is Associated With Incident Frailty in Older Men: The Concord Health and Aging in Men Project
Source: J Gerontol A Biol Sci Med Sci. 2022 Mar 30;77(9):1853–65. doi: 10.1093/gerona/glac077 (PMC9434472; doi:10.1093/gerona/glac077)
Supplement: glac077_suppl_Supplementary_Tables [file glac077_suppl_supplementary_tables.pdf]

Supplementary Table 1. Cross-sectional associations between dietary iron intakes and FI frailty status (n = 758)

**Iron intake**	Low tertile (reference category)	Medium tertile	High tertile	As continuous variable
Total iron[a]				
Model 1	1	0.93 (0.46, 1.35) *p* = .70	0.77 (0.53 ,1.13) *p* = .19	0.98 (0.96, 1.01) *p* = .13
Model 2	1	1.15 (0.74, 1.81) *p* = .53	1.02 (0.60, 1.74) *p* = .95	0.98 (0.96, 1.00) *p* = .10
Model 3				
Without haemoglobin	1	1.10 (0.61, 1.98) *p* = .77	1.23 (0.61, 2.47) *p* = .56	0.99 (0.96, 1.01) *p* = .29
With haemoglobin	1	1.07 (0.59, 1.95) *p* = .83	1.18 (0.58, 2.39) *p* = .64	0.98 (0.96, 1.01) *p* = .23
Haem iron[b]				
Model 1	1	0.80 (0.55, 1.17) *p* = .25	0.81 (0.55, 1.18) *p* = .26	0.91 (0.77, 1.07) *p* = .23
Model 2	1	0.99 (0.65, 1.53) *p* = .98	0.98 (0.60, 1.60) *p* = .94	0.90 (0.72, 1.13) *p* = .35
Model 3				
Without haemoglobin	1	1.068 (0.06, 1.87)	1.07 (0.57, 2.01) *p* = .84	0.93 (0.69, 1.26) *p* = .64

		p = .85		
With haemoglobin	1	1.08 (0.61, 1.92)<br>*p* = .79	1.13 (0.60, 2.16)<br>*p* = .70	0.95 (0.70, 1.29)<br>*p* = .76
Non-haem iron[c]				
Model 1	1	0.97 (0.67, 1.40)<br>*p* = .85	0.79 (0.54, 1.15)<br>*p* = .22	0.97 (0.93, 1.01)<br>*p* = .094
Model 2	1	1.21 (0.78, 1.89)<br>*p* = .40	1.03 (0.60, 1.87)<br>*p* = .92	0.96 (0.91, 1.02)<br>*p* = .19
Model 3				
Without haemoglobin	1	1.15 (0.63, 2.10)<br>*p* = .65	1.07 (0.52, 2.18)<br>*p* = .86	0.96 (0.89, 1.04)<br>*p* = .34
With haemoglobin	1	1.09 (0.59, 2.00)<br>*p* = .78	1.00 (0.49, 2.06)<br>*p* = 1.00	0.95 (0.88, 1.03)<br>*p* = .24
Haem to non-haem iron ratio %[d]				
Model 1	1	1.35 (0.92, 1.97)<br>*p* = .12	1.24 (0.85, 1.82)<br>*p* = .27	0.99 (0.98, 1.01)<br>*p* = .48
Model 2	1	1.45 (0.94, 2.24)<br>*p* = .096	1.23 (0.77, 1.97)<br>*p* = .39	0.99 (0.97, 1.01)<br>*p* = .42
Model 3				
Without haemoglobin	1	1.69 (0.95, 3.01)<br>*p* = .073	1.26 (0.67, 2.40)<br>*p* = .48	1.00 (0.97, 1.03)<br>*p* = .90
With haemoglobin	1	1.73 (0.97, 3.10)<br>*p* = .064	1.38 (0.72, 2.64)<br>*p* = .34	1.00 (0.97, 1.03)<br>*p* = .88

Notes: Model 1 unadjusted (n = 758 for total, 234 frail); Model 2 adjusted by sociodemographic and lifestyle factors (age, BMI, country of birth, marital status, age pension, alcohol consumption, smoking status, energy intake, Australian Dietary Guideline Index, number of serves of fruits, vegetables, grains, meat/alternatives, dairy/alternatives, iron and/or multivitamin supplement use) (n = 741 for total, 226 frail); Model 3 adjusted by Model 2 plus health (IL-6, NSAID and/or PPI use, self-rated health and number of comorbidities) without haemoglobin (n = 699 for total, 212 frail) and with haemoglobin (n = 694 for total, 212 frail)

[a] Low tertile ≤11.34mg/d, n = 253 with median (IQR) 9.54 (8.17, 10.56); medium tertile 11.35-14.91mg/d, n = 253 with median (IQR) 12.97 (12.10, 13.93); high tertile ≥14.92mg/d, n = 252 with median (IQR) 17.73 (15.97, 20.16)

[b] Low tertile ≤1.38mg/d, n = 253 with median (IQR) 1.00 (0.72, 1.20); medium tertile 1.39-2.14mg/d, n = 253 with median (IQR) 1.74 (1.56, 1.93); high tertile ≥2.15mg/d, n = 252 with median (IQR) 2.71 (2.41, 3.20)

[c] Low tertile ≤9.58mg/d, n = 253 with median (IQR) 8.00 (6.67, 8.79); medium tertile 9.59-12.77mg/d, n = 253 with median (IQR) 11.04 (10.38, 11.86); high tertile ≥12.78mg/d, n = 252, with median (IQR) 15.40 (13.83, 17.69)

[d] Low tertile ≤11.92%/d, n = 253 with median (IQR) 8.61 (6.20, 10.27); medium tertile 11.93-19.39%/d, n = 253 with median (IQR) 15.34 (13.85, 17.53); high tertile ≥19.40%/d, n = 252 with median (IQR) 25.53 (22.37, 32.01)

Supplementary Table 2. Median (interquartile range) daily dietary iron intakes and changes in dietary iron intakes according to transitions in FP frailty status (n=563)

Transition from baseline to 3-year follow-up	Robust (n=293)			Pre-frail (n=270)		
	Robust (n=124)	Pre-frail (n=154)	Frail (n=15)	Robust (n=22)	Pre-frail (n=177)	Frail (n=71)
Total iron mg/d						
Baseline	14.04 (11.55, 17.04)	12.67 (10.25, 15.06)	13.81 (11.38, 18.01)	12.52 (10.32, 15.39)	12.71 (10.55, 15.92)	14.12 (11.28, 17.16)
3-year follow-up	12.77 (10.38, 15.85)	11.58 (9.49, 14.73)	12.63 (9.63, 14.59)	13.48 (9.03, 16.81)	12.19 (10.01, 14.89)	12.26 (10.57, 14.40)
Change	-0.78 (-3.69, 1.67)	-0.94 (-3.15, 1.96)	-2.27 (-4.05, 0.67)	0.06 (-2.61, 2.91)	-0.67 (-3.50, 1.40)	-1.51 (-4.98, 1.68)
Haem iron mg/d						
Baseline	1.78 (1.22, 2.33)	1.72 (1.14, 2.39)	1.78 (1.16, 2.63)	1.78 (1.18, 2.94)	1.78 (1.25, 2.47)	1.66 (1.14, 2.25)
3-year follow-up	1.57 (1.13, 2.05)	1.53 (1.07, 2.03)	1.18 (0.79, 1.61)	1.84 (1.17, 2.63)	1.61 (1.04, 2.16)	1.32 (0.93, 1.93)
Change	-0.02 (-0.74, 0.45)	-0.07 (-0.75, 0.39)	-0.61 (-1.15, 0.02)	0.09 (-1.01, 0.61)	-0.11 (-0.88, 0.38)	-0.16 (-0.83, 0.40)

Non-haem iron mg/d						
Baseline	11.83 (10.14, 14.83)	10.89 (8.55, 12.92)	10.91 (9.60, 15.96)	10.39 (7.94, 13.60)	10.86 (8.80, 13.20)	12.10 (9.77, 15.27)
3-year follow-up	11.19 (9.00, 14.34)	9.94 (7.88, 12.28)	11.33 (8.59, 13.60)	11.08 (8.08, 13.56)	10.44 (8.66, 12.96)	10.14 (8.96, 12.37)
Change	-0.74 (-2.96, 1.39)	-0.73 (-2.63, 1.79)	-1.17 (-3.45, 0.54)	0.36 (-1.78, 1.55)	-0.36 (-2.58, 1.54)	-1.47 (-4.93, 1.20)
Haem to non-haem iron ratio %/d						
Baseline	13.93 (9.70, 21.01)	15.55 (10.87, 23.88)	12.83 (8.88, 22.64)	16.52 (10.56, 27.22)	15.23 (10.61, 22.61)	13.29 (8.75, 19.39)
3-year follow-up	14.32 (10.01, 19.10)	15.89 (10.34, 21.15)	11.89 (7.54, 16.02)	19.16 (9.71, 25.43)	15.06 (10.58, 20.78)	12.83 (8.81, 21.04)
Change	0.25 (-4.46, 4.31)	-0.75 (-6.52, 5.07)	-2.42 (-8.07, 2.87)	-0.76 (-6.53, 5.57)	-0.29 (-6.68, 5.46)	-0.24 (-5.15, 4.24)

Supplementary Table 3. Median (interquartile range) daily dietary iron intakes and changes in dietary iron intakes according to transitions in FI frailty status (n=432)

Transition from baseline to 3-year follow-up	Non-frail (n=432)	
	Non-frail (n=198)	Frail (n=234)
Total iron mg/d		
Baseline	12.76 (10.61, 15.59)	13.60 (10.67, 16.79)
3-year follow-up	12.06 (9.95, 14.49)	11.94 (9.92, 15.19)
Change	-1.18 (-3.45, 1.58)	-1.18 (-4.22, 1.71)
Haem iron mg/d		
Baseline	1.77 (1.28, 2.39)	1.73 (1.12, 2.42)
3-year follow-up	1.57 (1.04, 2.15)	1.45 (1.09, 2.05)
Change	-0.17 (-0.94, 0.45)	-0.07 (-0.71, 0.31)
Non-haem iron mg/d		
Baseline	10.83 (8.84, 13.51)	11.64 (9.00, 14.51)
3-year follow-up	10.25 (8.77, 12.19)	10.44 (8.51, 13.35)
Change	-0.70 (-3.16, 1.46)	-0.82 (-3.01, 1.65)
Haem to non-haem iron ratio %/d		
Baseline	15.49 (10.61, 23.89)	14.50 (9.68, 19.73)
3-year follow-up	15.58 (10.50, 20.93)	14.50 (10.08, 20.08)
Change	-0.39 (-6.80, 4.22)	-0.26 (-5.36, 5.05)

Supplementary Table 4. Longitudinal associations between changes in dietary iron intakes and incident FI frailty (n = 432)

**Iron intake**	Low tertile (reference category)	Medium tertile	High tertile	As continuous variable
Total iron[a]				
Model 1	1	0.97 (0.61, 1.55) *p* = .91	0.95 (0.60, 1.50) *p* = .81	0.98 (0.95, 1.01) *P* = 0.10
Model 2	1	0.94 (0.56, 1.57) *p* = .82036	1.15 (0.69, 1.93) *p* = .59	0.98 (0.96, 1.01) *p* = .14
Model 3				
Without haemoglobin	1	1.04 (0.59, 1.83) *p* = .89	1.21 (0.68, 2.15) *p* = .52	0.99 (0.96, 1.02) *p* = .44
With haemoglobin	1	0.97 (0.55, 1.73) *p* = .93	1.20 (0.67, 2.15) *p* = .54	0.99 (0.96, 1.02) *p* = .42
Haem iron[b]				
Model 1	1	1.22 (0.77, 1.94) *p* = 0.41	1.06 (0.67, 1.68) *p* = .81	1.06 (0.88, 1.27) *p* = .55
Model 2	1	1.11 (0.66, 1.85) *p* = .70	1.02 (0.61, 1.71) *p* = .94	1.06 (0.87, 1.30) *p* = .56
Model 3				
Without haemoglobin	1	1.04 (0.56, 1.92) *p* = .90	0.77 (0.41, 1.46) *p* = .43	0.90 (0.69, 1.17) *p* = .43
With haemoglobin	1	1.04 (0.65, 1.94)	0.78 (0.41, 1.49)	0.90 (0.68, 1.19)

		p = .90	*p* = .45	*p* = .45
Non-haem iron[c]				
Model 1	1	1.03 (0.65, 1.64) *p* = .91	0.97 (0.61, 1.55) *p* = .91	0.99 (0.95, 1.03) *p* = .065
Model 2	1	1.08 (0.65, 1.80) *p* = .77	1.17 (0.70, 1.96), *p* = .54	1.02 (0.96, 1.07) *p* = .57
Model 3				
Without haemoglobin	1	1.28 (0.71, 2.32) *p* = .41	1.44 (0.79, 2.63) *p* = .24	1.05 (0.98, 1.12) *p* = .18
With haemoglobin	1	1.22 (0.67, 2.21) *p* = .52	1.42 (0.78, 2.60) *p* = .25	1.04 (0.98, 1.12) *p* = .21
Haem to non-haem iron ratio %[d]				
Model 1	1	1.00 (0.63,1.59) *p* = 1.00	1.18 (0.74, 1.88) *p* = .48	1.01 (0.99, 1.03) *p* = .23
Model 2	1	0.94 (0.57, 1.56) *p* = .82	1.01 (0.61, 1.68) *p* = .98	1.01 (0.99, 1.03) *p* = .63
Model 3				
Without haemoglobin	1	0.69 (0.37, 1.27) *p* = .23	0.61 (0.32, 1.17) *p* = .14	0.98 (0.95, 1.01) *p* = .16
With haemoglobin	1	0.72 (0.39, 1.34) *p* = .30	0.65 (0.34, 1.25) *p* = .20	0.98 (0.95, 1.01) *p* = .16

Notes: Model 1 unadjusted (n = 432 for total, 234 frail); Model 2 adjusted by sociodemographic and lifestyle factors (age, BMI, country of birth, marital status, age pension, alcohol consumption, smoking status, energy intake, Australian Dietary Guideline Index, number of

serves of fruits, vegetables, grains, dairy/alternatives and meat/alternatives, iron and/or multivitamin supplement use) (n = 426 for total, 233 frail); Model 3 adjusted by Model 2 plus health and respective baseline iron intake (IL-6, NSAID and/or PPI use, self-rated health, number of comorbidities and respective baseline iron intake) without haemoglobin (n = 402 for total, 219 frail) and with haemoglobin (n = 398 for total, 216 frail)

[a] Low tertile ≤-2.83mg/d, n = 144 with median (IQR) -5.09 (-7.62, -3.92); medium tertile -2.82-0.72mg/d, n = 144 with median (IQR) -1.18 (-2.07, -0.15); high tertile ≥0.73mg/d, n = 144 with median (IQR) 3.03 (1.66, 5.20)

[b] Low tertile ≤-0.53mg/d, n = 144 with median (IQR) -1.11 (-1.59, -0.82), medium tertile -0.52-0.18mg/d, n = 144 with median (IQR) -0.11 (-0.38, 0.04), high tertile ≥0.19mg/d, n = 144 with median (IQR) 0.64 (0.37, 1.06)

[c] Low tertile ≤-2.25mg/d, n = 144 with median (IQR) -4.46 (-6.39, -3.07), medium tertile -2.24-0.73mg/d, n = 144 with median (IQR) -0.77 (-1.45, 0.04), high tertile ≥0.74mg/d, n = 144 with median (IQR) 2.67 (1.53, 4.74)

[d] Low tertile ≤-4.01%/d, n = 144 with median (IQR) -9.16 (-15.25, -5.79), medium tertile -4.00-3.08%/d, n = 144 with median (IQR) -0.31 (-1.83, 1.30), high tertile ≥3.09%/d, n = 144 with median (IQR) 6.90 (4.75, 10.93)
